# Evaluation of a mental health service reform program, the Pathways to Community Living Initiative, for people with severe mental illness and complex needs

**DOI:** 10.1177/10398562231211673

**Published:** 2023-11-15

**Authors:** Kathryn E Williams, Conrad Kobel, Anita Westera, Peri O’Shea, Cristina Thompson, Kate Jackson, Robyn Murray

**Affiliations:** Australian Health Services Research Institute, 8691 University of Wollongong, Wollongong, NSW, Australia; Australian Health Services Research Institute, 8691 University of Wollongong, Wollongong, NSW, Australia; Australian Health Services Research Institute, 8691 University of Wollongong, Wollongong, NSW, Australia; Australian Health Services Research Institute, 8691 University of Wollongong, Wollongong, NSW, Australia; Australian Health Services Research Institute, 8691 University of Wollongong, Wollongong, NSW, Australia; NSW Ministry of Health, St Leonards, NSW, Australia; NSW Ministry of Health, St Leonards, NSW, Australia

**Keywords:** evaluation, health service reform, severe mental illness, rehabilitation, deinstitutionalisation

## Abstract

**Objective:**

The Pathways to Community Living Initiative (PCLI) aims to reform mental health care for people with severe and persistent mental illness (SPMI) and complex needs. This study reports independent evaluation findings on transitions from hospital and practice change in mental health services.

**Methods:**

Data for this mixed-methods evaluation were obtained from administrative collections and semi-structured interviews with PCLI program managers, teams and executive leads; aged care managers; and leaders in inpatient, community and older people’s mental health services.

**Results:**

Between July 2015 and December 2020, 674 participants (67% of those eligible for the PCLI) were transitioned from hospital to community. Of those transitioned, 21 required subsequent long-stay admissions. The PCLI introduced resources, clearly defined processes, and state-wide networks to guide changes in practice which are becoming embedded in the operations and governance of mental health services across New South Wales.

**Conclusions:**

Severe and persistent mental illness and complex needs can be managed in community settings with highly individualised planning and care, supported by specialised clinical teams in partnership with mental health, aged care and disability services. Evaluation findings highlight the importance of continued investment in rehabilitation psychiatry.

People with severe and persistent mental illness (SPMI) and complex needs are at risk of long hospitalisation.^1,2^ These patients are a ‘low volume, high need, high-cost group’^3^ with little evidence-based guidance for treatment.^4^ Many have a primary diagnosis of psychosis. Their severe, treatment-resistant symptoms, combined with physical illness, disabilities, cognitive impairment, and/or substance misuse, affect all aspects of daily functioning.

Profound and lasting change in the treatment of people with SPMI cannot be achieved in a piecemeal fashion by services but needs to be driven and coordinated at the highest levels.^5^ As a major component of the New South Wales (NSW) mental health reform strategy,^5^ the Pathways to Community Living Initiative (PCLI) aims to address challenges in psychiatric rehabilitation and overcome barriers for these patients. Design and implementation processes (Figure 1) were informed by consultation^6^ and evidence.^7,8^

**Figure 1.**
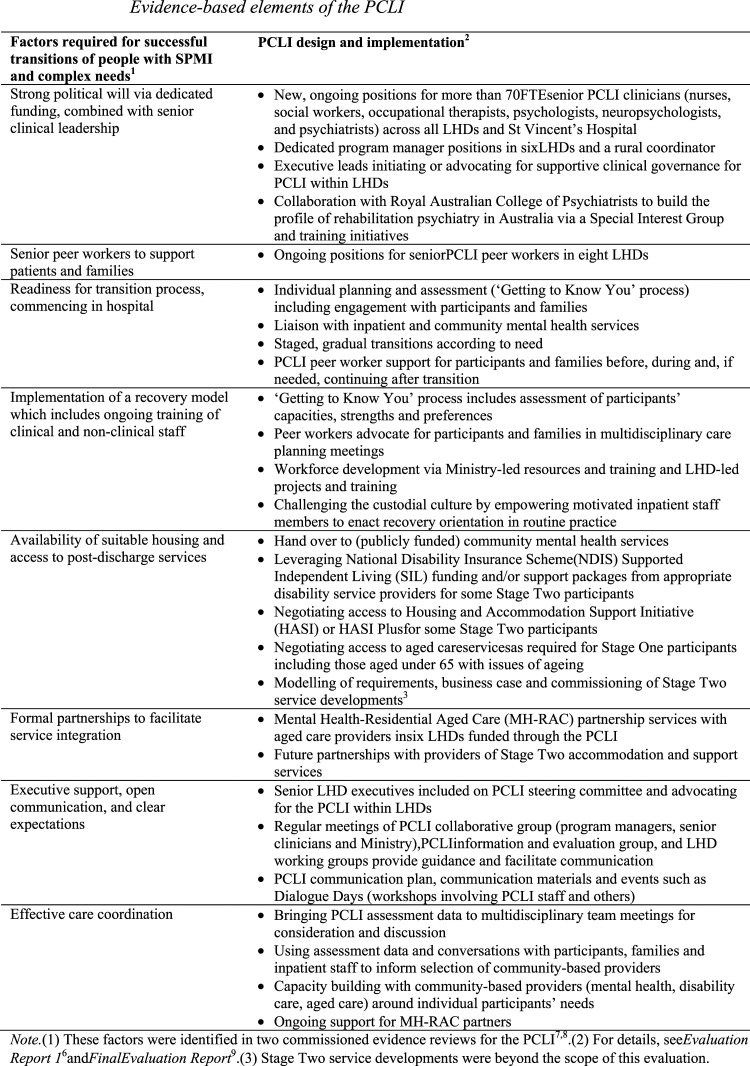
Evidence-based elements of the PCLI.

An independent evaluation of the PCLI^9^ was conducted between 2017 and 2021. This article focuses on two evaluation questions: first, whether the PCLI succeeded in transitioning participants out of hospital, and second, whether the program influenced practice in mental health services to decrease the likelihood of future long hospital stays across the population of people with SPMI and complex needs.

## Methods

The PCLI is funded and driven by the NSW Ministry of Health (the Ministry) in cooperation with Local Health Districts (LHDs). The program began in mid-2015 with recruitment of program managers in six LHDs where large, non-acute facilities were situated and later expanded to all LHDs. Initially, the target population was people who had been inpatients for 365 days or longer; this was later expanded to those at risk of long stays. There are two PCLI cohorts, defined by functioning: adults with issues of ageing such as dementia or frailty (Stage One) and adults without issues of ageing (Stage Two). Five LHDs have Stage One and Stage Two PCLI teams, one LHD has a Stage One team only and at the remaining LHDs, Stage Two is the focus of PCLI activities.

### Evaluation design

The mixed-methods evaluation was designed around an established framework^10^ and had three components, which all received ethical approval. Two are reported here: patient outcomes and provider/system change. The third, patient experiences, is reported elsewhere in this issue.^11^

### Participants

The patient outcomes evaluation used administrative data for the entire PCLI cohort (Table 1), that is, all patients eligible for the program. The provider/system change evaluation included 167 semi-structured interviews, conducted over 5 years, with PCLI program managers, clinical teams and executive leads; aged care managers; and leaders in inpatient, community and older people’s mental health services. The staff of mental health and aged care services who were interviewed for this study were carefully chosen (purposive sampling) because of their long exposure to, and deep knowledge of, the program and the patients who took part.

**Table 1.** Profile of PCLI cohort.^a^

Characteristic	Stage One (*N* = 227)		Stage Two (*N* = 777)		All PCLI (*N* = 1004)	
	*n*	%	*n*	%	*n*	%
Sex						
Male	138	60.8	531	68.3	669	66.6
Female	89	39.2	246	31.7	335	33.4
Age group						
Younger than 25	0	0.0	44	5.7	44	4.4
25–34	2	0.9	148	19.0	150	14.9
35–44	4	1.8	212	27.3	216	21.5
45–54	16	7.0	209	26.9	225	22.4
55–64	55	24.2	152	19.6	207	20.6
65–74	100	44.1	11	1.4	111	11.1
75–84	42	18.5	1	0.1	43	4.3
85 and over	8	3.5	0	0.0	8	0.8
Principal diagnosis						
Schizophrenia	86	37.9	433	55.7	519	51.7
Schizoaffective disorders	33	14.5	114	14.7	147	14.6
Dementia in Alzheimer's disease	17	7.5	0	0.0	17	1.7
Vascular or unspecified dementia or dementia in other disease	17	7.5	2	0.3	19	1.9
Bipolar affective disorder	12	5.3	7	0.9	19	1.9
All other diagnoses	25	11.0	40	5.1	65	6.5
Missing^b^	37	16.3	181	23.3	218	21.7
Total length of stay^c^						
Less than 1 year	58	25.6	175	22.5	233	23.2
1–2 years	59	26.0	243	31.3	302	30.1
2–3 years	28	12.3	138	17.8	166	16.5
3–4 years	13	5.7	67	8.6	80	8.0
4–5 years	10	4.4	35	4.5	45	4.5
5–6 years	10	4.4	21	2.7	31	3.1
6–7 years	15	6.6	14	1.8	29	2.9
7–8 years	2	0.9	6	0.8	8	0.8
8–9 years	3	1.3	15	1.9	18	1.8
9–10 years	10	4.4	13	1.7	23	2.3
10 or more years	19	8.4	50	6.4	69	6.9

^a^The PCLI cohort was defined as people for whom entries existed in the PCLI database, that is, people who had been identified by LHD staff as having experienced, or at risk of experiencing, long hospital stays. The cut-off date for inclusion was 31 December 2020.

^b^Diagnosis coding and DRG classification only occurs after the inpatient episode has concluded. Therefore, there was no diagnostic or DRG information available for ongoing or recently concluded stays.

^c^Length of stay is reported as it is recorded in the Health Information Exchange. This does not include any previous stays. Length of stay for consumers who remained in hospital was calculated to 31 December 2020.

### Materials

Patient outcomes were measured with routine tools^12^: Kessler Psychological Distress Scale (K10,^13^ patient rating of global distress); Health of the Nation Outcome Scales (HoNOS, HoNOS65+^14^; severity of mental illness symptoms); Life Skills Profile (LSP-16,^15^ quality of life and general functioning) and Resource Utilisation Groups – Activities of Daily Living (RUG-ADL,^16^ functional independence in four domains: bed mobility, toileting, transfer and eating). Interview schedules were designed around the evaluation questions and focused on implementation (e.g. barriers and enablers to program delivery) and observed outcomes (e.g. changes in practice in mental health services, outcomes for patients who were transitioned to community living).

### Procedure

De-identified administrative data were provided to the evaluation team from the Ministry. Data included patient demographics, inpatient stay characteristics, use of health services following transition and scores on the outcome measures before and after discharge.

Interviews took place with individuals or groups at 11 implementation sites and nine aged care facilities. Some individuals were interviewed on multiple occasions, providing a longitudinal perspective. Participants were provided with written information and gave written or verbal consent.

### Analysis

The discharge date from the index stay – the admission that ended in transition to community – was identified for each participant and used as the reference point for subsequent health service use and outcome measures. For participants who had not yet transitioned, the index stay was the ongoing admission. Only valid, complete assessments were retained. Paired t-tests and Wilcoxon signed rank tests were used to examine differences between baseline and follow-up scores on the outcome measures for those who transitioned.

Interview transcripts were managed in NVivo 12 Plus. Three evaluators, each with extensive experience of qualitative analysis and lengthy exposure to the PCLI, worked collaboratively to code, index and analyse data. They used modified framework analysis^17^ to achieve a holistic, descriptive overview and iterative categorisation^18^ to move systematically from codes and themes to interpretation.

## Results

### Transitions to community

Two-thirds of the PCLI cohort (674, 67%) were transitioned to the community by the end of December 2020 (Table 2). After transition there was no change in distress (K10) and mental health did not deteriorate significantly (HoNOS ‘symptom’, ‘behaviour’ and ‘social’ sub-scales and total scores; Table 3). General functioning and quality of life (LSP-16) improved on average for Stage One, but there was significant decline in HoNOS 65+ ‘impairment’ and in RUG-ADL scores for all domains except toileting. Stage Two showed deterioration in cognition and physical health (HoNOS ‘impairment’ sub-scale) and general functioning and quality of life (LSP-16). Nevertheless, clinicians and aged care managers reported that they had observed benefits of transition for many participants, including improved quality of life, functional independence, and social participation, and better mental and physical health.

**Table 2.** Transition outcomes for PCLI cohort to 31 December 2020.

Outcome	Stage One (*N* = 227)		Stage Two (*N* = 777)		All PCLI (*N* = 1004)	
	*n*	%	*n*	%	*n*	%
Discharged to community living	156	68.7	518	66.7	674	67.1
Remained in hospital	43	18.9	252	32.4	295	29.4
Died in hospital	28	12.3	7	0.9	35	3.5

**Table 3.** Health outcomes following transition.

Outcome tools	Stage One (*N* = 156)				Stage Two (*N* = 518)			
	Pairs (*n*)	Baseline mean (SD)	Follow-up mean (SD)	Mean difference	Pairs (*n*)	Baseline mean (SD)	Follow-up mean (SD)	Mean difference
K10	50	20.8 (9.0)	17.8 (8.2)	−2.9	308	16.2 (7.7)	16.1 (7.6)	−0.1
LSP-16								
Withdrawal	80	47.3 (26.2)	47.8 (24.2)	0.5	350	30.4 (21.5)	35.9 (22.6)	5.4 **
Self-care	80	53.7 (23.5)	47.9 (24.4)	−5.7 *	350	34.7 (19.7)	36.7 (18.9)	2.0 *
Compliance	80	33.6 (28.8)	26.5 (22.3)	−7.1 *	350	19.0 (19.0)	24.0 (21.6)	5.0 **
Antisocial	80	32.5 (27.7)	22.9 (21.8)	−9.6 **	350	17.6 (19.4)	17.6 (19.9)	0.0
Total	80	43.0 (22.4)	37.6 (18.7)	−5.4 *	350	26.4 (15.7)	29.3 (16.2)	2.9 **
HoNOS								
Behaviour	47	8.9 (11.8)	9.8 (12.0)	0.9	458	8.7 (12.2)	8.5 (12.1)	−0.2
Impairment	48	34.4 (21.2)	35.7 (22.9)	1.3	468	18.2 (19.4)	23.4 (18.7)	5.0 *
Symptom	44	25.0 (13.8)	28.2 (19.7)	3.2	457	19.2 (18.3)	20.7 (19.0)	1.5
Social	45	28.9 (23.0)	26.1 (16.6)	−2.8	439	22.3 (22.5)	22.0 (19.2)	−0.3
Total	41	23.5 (13.5)	24.8 (12.0)	1.3	427	17.4 (15.2)	18.4 (13.5)	1.1
HoNOS 65+								
Behaviour	84	10.8 (10.2)	9.5 (8.5)	−1.2				
Impairment	84	47.6 (28.4)	55.8 (28.9)	8.2*				
Symptom	79	23.9 (15.9)	25.7 (16.9)	1.8				
Social	76	32.0 (19.7)	29.5 (16.6)	−2.5				

*Note.* * indicates significant at *p < .05* and ** significant at *p < .001*. These *p*-values refer to the paired t-tests or Wilcoxon Signed Rank tests (non-parametric equivalent) used to examine differences between the baseline and the follow-up assessment. Sub-scale scores were standardised to represent percentage of maximum possible score to allow comparisons. Lower scores indicate less severe problems. HoNOS 65+ was used with people aged 65 years and over. RUG-ADL has a different scoring system and is not shown here; it was used only with Stage One.

About 20% of Stage One and 33% of Stage Two participants were readmitted to mental health units. Most readmissions resulted in stays of 4 weeks or less (Table 4). A small proportion (7–8%) presented to emergency departments (ED). For Stage One, most presentations to ED were not mental health related. Three-quarters of Stage Two presentations were accounted for by one percent (9/777) of individuals. Most participants had at least one contact with mental health community services. Contacts occurred, on average, every 23 days for Stage One and every 8 days for Stage Two.

**Table 4.** Health service use following transition.

Service type	Characteristics	Stage One (*N* = 156)		Stage Two (*N* = 518)	
		*n*	%	*n*	%
Inpatient	Persons				
	At least one readmission	69	44.2	218	42.1
	Long stay (365+ days)	3	2.0	18	3.5
	At risk of long stay (180–364 days)	3	2.0	7	1.4
	Readmissions				
	Mental health related	68	7.1	695	76.4
	Non mental health related	892	92.9	215	23.6
	All readmissions	960	100.0	910	100.0
Emergency department	Persons				
	At least one presentation	11	7.1	39	7.5
	Presentations				
	Primary mental health diagnosis	2	6.1	76	37.8
	Other primary diagnosis	31	93.9	125	62.2
	All presentations	33	100.0	201	100.0
Community mental health	Persons				
	At least one contact	140	89.7	497	95.9
	Contacts				
	All contacts	5707	100.0	46,224	100.0

### Practice change in mental health services

The PCLI introduced clearly defined processes to guide practice change. These included assessment of goals, capacities and support needs, careful matching with suitable community-based providers and capacity building – tailored to each participant – with providers and community mental health services. Significant investment, including funding for aged care partnerships and strengthened community mental health teams, provided a foundation for implementing reform. The PCLI senior clinicians assisted with transition planning, built capacity within mental health care teams, and supported organisations providing aged care and disability care services to participants. PCLI peer workers supported patient engagement and were strong advocates within multidisciplinary teams. State-wide networks enabled common training for PCLI teams, facilitating trouble-shooting and problem-solving across teams. Regular network meetings facilitated implementation and quality control and supported fidelity to PCLI processes.

By the end of the evaluation period, most LHDs had embedded fundamental components of the PCLI within operations and clinical governance. Practice change was occurring; however, services still required extensive input from PCLI teams through monitoring and capacity building.

Interviewees pointed out that as more patients are transitioned out of hospital, there will be a greater need for services from community mental health teams, because the ongoing clinical care and behavioural support that some people require is not available via aged care services or disability services. Consequently, stakeholders believe that sustainability of the PCLI will depend on addressing resourcing issues in publicly funded community mental health care, maintaining clarity around roles and responsibilities, and continuing to build capacity among staff of aged care and disability services so they can better support people with SPMI and complex needs.

## Discussion

During the evaluation period, two-thirds of people eligible for the PCLI were transitioned to community living. Despite their ongoing, severe mental illness and complex needs, the vast majority did not require subsequent long-stay hospital admissions. Most participants received clinical follow-up in the community, and mental health crises requiring ED attendance were rare. On average, there was no increase in distress and no exacerbation of mental health symptoms. Many became more sociable and better able to care for themselves and had improved mental and physical health and quality of life, according to the mental health clinicians and aged care managers who observed them. The outcomes data indicated that those with issues of ageing had, on average, significant increases in life skills but decreases in independent functioning, which may be explained by normal ageing processes. Declines for Stage Two in general functioning and quality of life were more difficult to interpret but may be due to sampling bias, as explained below.

The qualitative evaluation of provider/system change shed light on how outcomes were achieved. The PCLI challenged mental health services to avoid long stays and to manage risks associated with discharge. It equipped them to unpack the capacities and needs of people with SPMI and assemble appropriate supports. Successful transitions relied on individualised planning and tailored, integrated care, in partnership with community-based services.

### Limitations

The quantitative findings rely on the accuracy of data from mental health services. Sometimes a continuous period of hospitalisation is reported as multiple stays, so the true duration remains unknown. Program guidelines indicate that PCLI participants should be followed up, and the outcome tools administered, every 6 months for up to 2 years after transition, however, the extent of follow-up was variable. This may have created a biased sample for the post-transition assessments, that is, participants requiring more intensive support may have been more likely to have a follow-up assessment, whereas those functioning relatively well in the community may have had missing data at follow-up.

## Conclusions

Notwithstanding these limitations, the PCLI demonstrated that SPMI and complex needs can be managed in community settings when people receive tailored supports and services. Evaluation findings highlight the importance of ongoing investment in rehabilitation psychiatry in Australia.
